# New potential biomarkers of ulcerative colitis and disease course — integrated metagenomic and metabolomic analysis among Polish patients

**DOI:** 10.1007/s00535-025-02280-6

**Published:** 2025-07-04

**Authors:** Oliwia Zakerska-Banaszak, Karolina Ladziak, Dariusz Kruszka, Kacper Maciejewski, Lukasz Wolko, Iwona Krela-Kazmierczak, Agnieszka Zawada, Marie Vibeke Vestergaard, Agnieszka Dobrowolska, Marzena Skrzypczak-Zielinska

**Affiliations:** 1 Institute of Human Genetics Polish Academy of Science, Strzeszynska 32, 60-479 Poznan, Poland; 2 Department of Biometry and Bioinformatics, Institute of Plant Genetics Polish Academy of Sciences, Strzeszynska 34, 60-479 Poznan, Poland; 3 Department of Medical Biotechnology, Poznan University of Medical Sciences, Fredry 10, 61-701 Poznan, Poland; 4 Department of Biochemistry and Biotechnology, Poznan University of Life Sciences, Dojazd 11, 60-632 Poznan, Poland; 5 Department of Gastroenterology, Dietetics and Internal Diseases, Poznan University of Medical Sciences, Przybyszewskiego 49, 60-355 Poznan, Poland; 6 Center for Molecular Prediction of Inflammatory Bowel Disease, PREDICT, Department of Clinical Medicine, Aalborg University, A.C. Meyers Vænge 15, 2450 Copenhagen, Denmark

**Keywords:** Ulcerative colitis course, Gut microbiota, Metabolome

## Abstract

**Background & aim:**

The course of ulcerative colitis (UC) involves successive periods of remission and exacerbation but is difficult to predict. Gut dysbiosis in UC has already been intensively investigated. However, are periods of exacerbation and remission associated with specific disturbances in the composition of the intestinal microbiota and its metabolome? Our goal was to answer this question and to identify bacteria and metabolites necessary to maintain the remission.

**Methods:**

We enrolled 65 individuals, including 20 UC patients in remission, 15 in exacerbation, and 30 healthy controls. Metagenomic profiling of the gut microbial composition was performed based on 16S rRNA V1-V9 sequencing. Stool and serum metabolic profiles were studied by chromatography combined with mass spectrometry.

**Results:**

We revealed significant differences in the gut bacterial and metabolic composition between patients in active UC and those in remission, as well as in healthy controls. As associated with UC remission we have identified following bacteria: *Akkermansia*, *Agathobacter, Anaerostipes*, *Enterorhabdus*, *Coprostanoligenes*, *Colinsella*, *Ruminococcus*, *Subdoligranulum*, *Lachnoclostridium*, *Coriobacteriales*, *Erysipelotrichaceae*, and *Family XII*, and compounds – 1-hexadecanol, phytanic acid, squalene, adipic acid, cis-gondoic acid, nicotinic acid, tocopherol gamma, ergosterol and lithocholic acid. Whereas, in the serum lithocholic acid, indole and xanthine were found as potential candidates for biomarkers of UC remission.

**Conclusion:**

We have demonstrated that specific bacteria, metabolites, and their correlations could be crucial in the remission of UC among Polish patients. Our results provide valuable insights and a significant source for developing new hypotheses on host-microbiome interactions in diagnosis and course of UC.

**Graphical abstract:**

**Figure:**
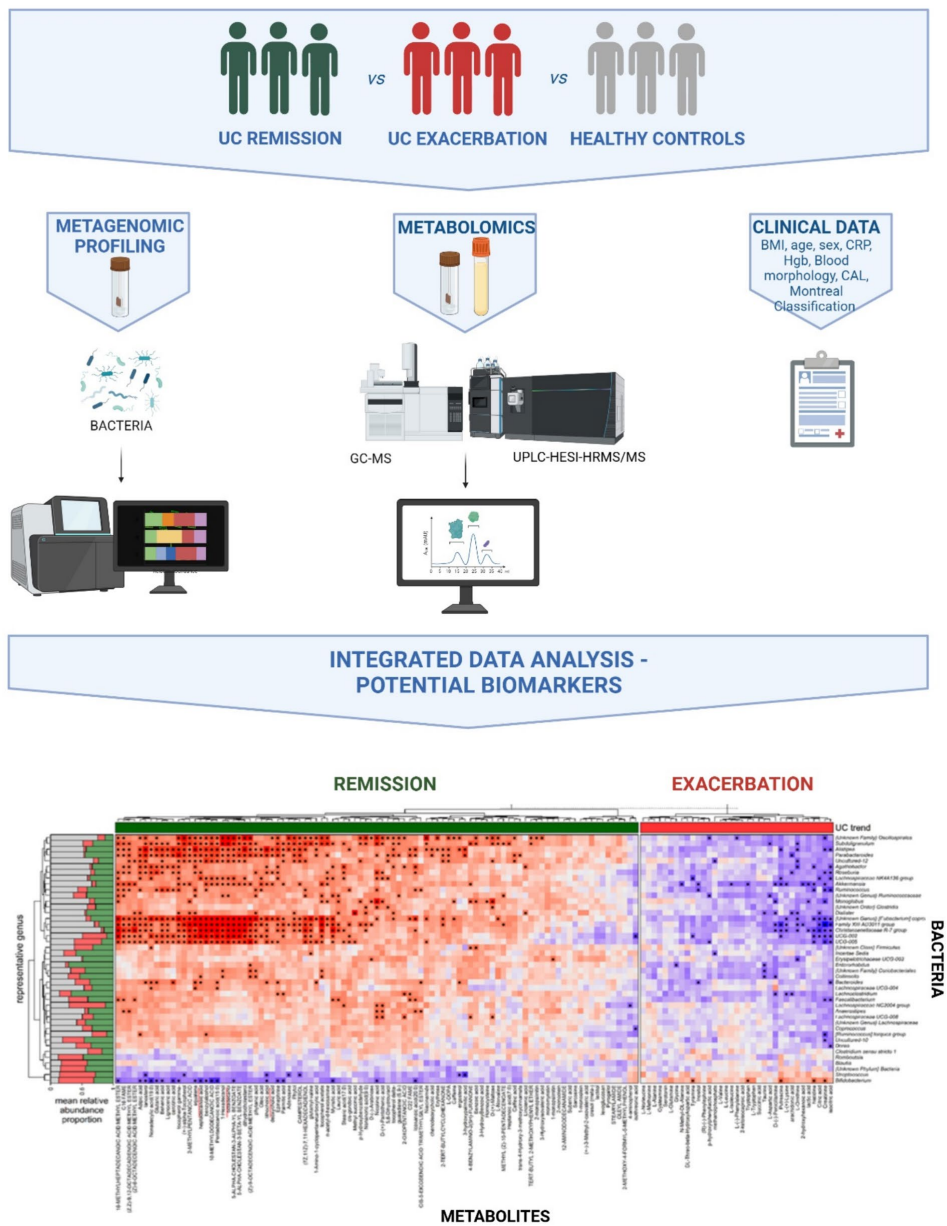
Created in BioRender. (2025) https://BioRender.com/3ut2ya0

**Supplementary Information:**

The online version contains supplementary material available at 10.1007/s00535-025-02280-6.

## Introduction

Ulcerative colitis (UC) is an inflammatory bowel disease (IBD) characterized by chronic, recurrent inflammation of the digestive tract with immuno-genetic and microbial origins, influenced by environmental factors [1, 2]. Its clinical course involving alternating periods of remission (R) and exacerbation (E) is often unpredictable [3]. Despite scientific advances, the cause of UC remains unclear. Over the past decade, UC incidence has increased worldwide with a further rise expected, highlighting the need for effective therapy or long-term remission [4].

Gut microbiota plays a key role in UC, as studies have shown that the intestinal microbiota from UC patients can induce the disease in germ-free mice [5–9]. UC is linked to dysbiosis, where microbial composition shifts, reducing biodiversity and increasing harmful bacteria (e.g., *Escherichia coli*, *Desulfovibrio*) while decreasing beneficial ones (e.g., *Lactobacillus*, *Akkermansia muciniphila*) what consequently alters the balance of gut Th17 and RORγt( +) regulatory T cells, disrupting the harmful inflammatory response reported among Polish patients in our previous study [10–14]. Research on microbial-derived metabolites, like butyrate, suggests their role in modulating the host epigenome, hormone release and mucosal integrity [15, 16]. There are myriad impacts of gut microbial compounds on host health; however, metabolomic studies have allowed us to discover only a small proportion of these effects [17, 18]. Understanding these metabolite changes may aid in UC diagnosis and treatment, though general microbial shifts for exacerbation are still unknown [19]. Therefore, functional studies on microbial metabolomics are so essential.

Our approach was to identify gut microbes and metabolites indicative of the clinical course of UC by quantitatively measuring microbiota-associated metabolites in fecal samples combined with metagenomic profiling. We hypothesized that specific bacterial and metabolomic signatures would significantly differ between UC patients during exacerbation and remission, compared to healthy controls. Thus, we aimed to characterize these signatures as potential biomarkers to enhance the prediction and management of UC flare-ups.

## Materials and methods

### Subjects and study design

The study was approved by the Ethics Committee of Poznan University of Medical Sciences (resolution no. 957/21 approved on 9 December 2021). All experiments were performed in accordance with the principles of the 1964 Declaration of Helsinki and its later amendments. All the participants provided written informed consent prior to their enrollment in the study. Thirty-five Polish patients with diagnosed UC (twenty patients in remission and fifteen in exacerbation of the disease) who were under the care of the University Clinical Hospital in Poznan and the Department of Gastroenterology, Dietetics and Internal Diseases at Poznan University of Medical Sciences in Poland were enrolled in this study. UC diagnosis was made based on clinical, endoscopic and histologic data. The exclusion criteria were additional diseases (e.g., *Clostridium difficile* infection, diabetes, or cancer), pregnancy and intake of antibiotics or probiotics during the last 3 months. The remission state was defined as at least 12 months of flare-free disease with clinical, histologic and endoscopic remission. The exacerbation state was defined as clinically active disease at the moment of involvement in the study.

The control group included thirty healthy Polish volunteers (eighteen women and twelve men) who fulfilled the following inclusion criteria: aged > 18 years, had a BMI between 18.5 and 25 years, did not have a diagnosis of gastrointestinal disorders or other serious diseases, did not smoke, had no regular alcohol intake, had no history of IBD in the family, and had no history of antibiotic or probiotic intake in the last 3 months.

For all individuals, UC patients and controls, the gut microbiota composition and fecal metabolites were determined.

### Sample collection

Fresh morning stool samples were taken to the sterile set as two aliquots (~ 1 g) by all participants and immediately frozen. The transfer of the samples to the laboratory was performed within 24 h of collection. All stool samples were stored at -80 °C until microbial DNA extraction. At the same day a peripheral blood sample (9 mL) were taken from each patient and serum was separated.

### Microbial community analysis

#### DNA extraction

Microbial DNA was isolated from 0.2 g of stool sample using a QIAmp Power Faecal Pro DNA Kit (QIAGEN, Germany) according to the manufacturer’s protocol. The concentration and purity of the extracted DNA were determined using a NanoDrop™ 2000 spectrophotometer (Thermo Fisher Scientific, Inc., Waltham, MA, USA). All DNA samples were stored at -20 °C until further molecular analysis.

#### Metagenomic library preparation and sequencing

Metagenomic libraries for next-generation sequencing (NGS) were prepared according to the QIAseq 16S/ITS Panel protocol (QIAGEN, Hilden, Germany) as we already described previously [8].

#### Metadata processing and bacterial taxa identification

The obtained sequences of the V1-V9 regions of the 16S rRNA gene were processed using CLC Genomic Workbench 11.0 with CLC Microbial Genomics Module 1.2 (Qiagen Bioinformatics, Aarhus, Denmark). In the first step, demultiplexing and trimming of the reads were performed. Next, alignment to the SILVA SSU 99% (v138.1) database of ribosomal RNA sequences was performed [20]. Chimeric sequences were removed, and operational taxonomic units (OTUs) for *Bacteria*, *Archaea* and *Eukarya* were distinguished. Paired reads were merged. The relative abundance of all identified microorganisms in the sample at each taxonomic level was calculated. The raw demultiplexed sequencing data with sample annotations were deposited in the Short Read Archive (SRA) database (PRJNA1040490).

Alpha diversity, based on OTU richness and Shannon entropy, as well as beta diversity was determined using a Bray‒Curtis dissimilarity matrix for the analyzed groups of samples.

For predicted metabolic pathway inside, the detected OTUs were subjected to PICRUSt2 tool.

### Metabolic profiling

#### Stool metabolite analysis with gas chromatography combined with mass spectrometry (GC‒MS)

A 0.1 g fecal sample was suspended in 1 mL of methanol and homogenized by vortexing for 1 h at 1400 rpm in a ThermoMixer (Eppendorf). The sample was then centrifuged for 10 min at 10,000 rpm at room temperature. The supernatant was pipetted into a new Eppendorf tube and centrifuged again. Finally, the supernatant was transferred to a fresh tube and dried in a centrifugal vacuum concentrator under vacuum and over P_2_O_5_. During derivatization, the polar groups in the sample were blocked. The derivatization procedure consisted of a 1.5-h incubation with 100 µL of methoxyamine hydrochloride in pyridine (20 mg/mL) at 37 °C, followed by a 30-min incubation with 160 µL of N-methyl-N-(trimethylsilyl)trifluoroacetamide (MSTFA) at 37 °C. The quality and quantity of all the samples were verified using a LECO Pegasus 4D system composed of a 7890A gas chromatograph (Agilent) and a LECO ToF mass analyser. LECO ChromaTOF software version 4.51.6.0 was used for data analysis. Gas chromatography was performed using a 30-m long, 0.25-mm internal diameter DB-5MS column with 0.25-µm film thickness (J&W Scientific, Agilent). For injection, a Gerstel CIS PTV-type injector was applied. The temperature of the injection was increased from 40 °C/sec to 240 °C, and the MS transfer line and ion source were set at 250 °C. Pure helium was used as the carrier gas at a constant flow rate of 1 mL/min. The oven temperature was held constant at 70 °C for 2 min, increased 10 °C/min to 300 °C, and held constant for 10 min at 300 °C. Mass spectra were recorded in the range of 35–650 m/z in EI + mode under standard 70 eV ionization conditions. The retention index mixture was analyzed prior to the relevant analyses, and an appropriate retention index method was created. Identification of peaks was performed based on their retention indices and comparisons of their spectra with those in proper mass spectra databases (NIST).

#### Detection and quantification of SCFAs with ultra-performance liquid chromatography coupled to high-resolution mass spectrometry (UPLC-HESI-HRMS/MS)

Fecal samples (0.1 g) were shaken with 1000 µL of 70% isopropanol (LC/MS grade, VWR) for 5 min at 4 °C on a vortex mixer (3000 rpm, IKA). The suspensions were homogenized for 10 min using an ultrasonic bath (Bandelin Sonorex) and centrifuged for 15 min (15,000 rpm, UNIVERSAL, Hettich) at 4 °C. Reagents: 30 µL of 45.0 mM 2-chloro-1-methylpyridinium iodide (Sigma Aldrich), 60 µL of 20-mM trimethylamine (Sigma Aldrich) and 200 µL of acetonitrile (ACN, LC‒MS grade, Sigma Aldrich) were added to 50 µL of the supernatant. The samples were incubated for 5 min at 50 °C (ThermoMixer C, Eppendorf). Next, the mixture was shaken with 100 µL of 45 mM 2-(diethylamino)ethanol (DEEA, Sigma Aldrich) for 40 min at 50 °C. The samples were dried using a speedvac system at 30 °C for 60 min. The dried samples were reconstituted in 300 µL of 50% ACN, centrifuged for 5 min (15,000 rpm) and transferred to chromatographic vials. The standard compounds were prepared in a similar way using a volatile free acid mixture (Supelco, CRM46975). The calibration curves were prepared for 0.01, 0.1, 1.0, 10, 100 and 1000 µM of each compound.

The parallel reaction monitoring (PRM) method was performed by applying ultraperformance liquid chromatography (Acquity, Waters) with high-resolution mass spectrometry (QExactive, Thermo Scientific). The sample (2 µL, partial loop mode) was separated on an Acquity HSS T3 column (2.1 × 50 mm, grain size 1.7 µm) at 30 °C using a gradient of 0.1% formic acid in water (LC‒MS grade – eluent A) and 0.05% formic acid in acetonitrile (eluent B) at a 300 µL min-1 flow rate. The linear gradient program was set as follows: initial to 1 min — 0.1% B, 7 min — 45% B, 9 min — 99% B, 11 min — isocratic 99% B, 12 min — 0.1% B and isocratic 0.1% B to 15 min. The HESI-II ion source was operated in positive ion mode. The parameters used were 3.5 kV — capillary voltages, 35 au — sheath gas flow, 10 au — auxiliary gas flow, 3 au —sweep gas flow and S-lens RF level 50. The ion transfer tube and auxiliary gas temperatures were set at 320 °C and 350 °C, respectively. The PRM settings were as follows: AGC-target, 5 × 104 ions; maximum injection time, 200 ms; isolation window, 1 m/z; and normalized collision energy, 25%.

#### Serum metabolite analysis

Samples — 100 µL of serum were extracted using 900 µL of acetonitrile (LC–MS grade, Merck, Darmstadt, Germany) with methanol (LC–MS grade, Merck, Darmstadt, Germany) 1:1 (v/v) in 1.5-mL tubes (Eppendorf, Hamburg, Germany) at 4 °C by 15 min. Tubes were centrifuged at 4 °C by 15 min using MIKRO 120 centrifuge (Hettich, Westphalia, Germany) and supernatants were transferred into HPLC-vials.

The untargeted metabolomics were performed using UPLC system (Acquity, Waters, Milford, MA, USA) with OrbiTrap based high resolution mass spectrometer (QExactive, Thermo Scientfic). The chromatography separation was performed using HSS T3 column (50 mm × 2.1 mm, 1.7 µm of grain size, Waters, Milford, MA, USA) at 30 °C. The gradient of water with 0.1% of formic acid (LC–MS grade, Merck, Darmstadt, Germany) and acetonitrile (LC–MS grade, Merck, Darmstadt, Germany) was 0.5% of B — 70% of B from 0 to 7 min, 70% of B — 99% of B from 7 to 9 min, isocratic 99% to 12 min and return to initial settings to 13 min at flow rate 0.4 mL/min. The eluate was ionizing using heated electrospray ion sources. HRMS operated in positive and negative ion mode at m/z range 75–1000. The FullMS scans were registered at resolution of 70 000 FWHM and 100 ms of maximum IT time. The data dependent MS/MS experiments were provided at resolution of 17 500 FWHM, 50 ms of maximum IT time and 30% of normalized collision energy.

The raw data were processed using MS-DIAL for peak extraction, annotation, LOESS normalization and export into.csv file [21]. The data post-processing was performed in the R environment using MSPrep package [22]. The data frames were filtrated based on sample/blank ratio (> fivefolds), number of missing values (< 30%) and relative standard deviation in QC group (RSD < 60). The missing values were estimated using 50% of minimum value of feature. The peak areas were scaled using log2.

### Statistical analyses

To analyze the differences in demographic data between groups, Student’s *t* test for independent measures was applied. For comparisons of metagenomic data, the Shapiro‒Wilk test was used first to determine whether the data were normally distributed, and if the assumptions were not met, the nonparametric Mann‒Whitney test was used. For three-group comparisons, a nonparametric multivariate statistical permutation test (PERMANOVA) or the Kruskal‒Wallis test with Dunn’s post hoc test was applied. The obtained peak areas of the metabolites were normalized and log-transformed. The data were imported into MStat for statistical analysis (http://proteom.ibb.waw.pl/mstat). The correlation analysis between metabolites and microbial taxa was performed with Spearman’s correlation with asymptotic t tests for P values. With respect to multiple hypothesis testing, the adjusted *P* values were calculated according to the Benjamini–Hochberg procedure. The statistical analysis was performed using STATA software (Stata statistical software: Release 15. College, TX: StataCorp LLC) and R 4.2.2 (R Foundation for Statistical Computing, Vienna, Austria). All tests were considered significant at *P* < 0.05. GraphPad Prism 8.0.1 (GraphPad Software, San Diego, CA, USA) and CLC Genomics Workbench software (Qiagen Bioinformatics, Aarhus, Denmark) were used to prepare the graphs.

## Results

### Subject characteristics

The study population included 65 participants, 30 HC and 35 UC patients, of which 20 (57%) were UC_R, while 15 (43%) UC_E. The detailed characteristics of all patients is presented in Table 1.

**Table 1:** Demographic characteristics of the studied subjects

Variable	UC patients		Healthy controls	*P* value
	(*n* = 35)		(*n* = 30)	
Female/Male, n (%)	19/16 (54.3/45.7)		18/12 (60/40)	0.642
Age, years (mean ± SD)	38.3 ± 12.1		35 ± 6.4	0.322
BMI, kg/m^2^ (mean ± SD)	22.2 ± 4.4		23.2 ± 3.1	0.38
Disease duration, years (mean ± SD)	8 ± 6.5			
	Remission (20)	Exacerbation (15)		
CRP, mg/L (mean ± SD)	3.7 ± 4.8	33.7 ± 48.8		< 0.01
Haemoglobin, g/dL (mean ± SD)	13.9 ± 1.4	8.8 ± 2.2		< 0.05
Calprotectin, µg/g (mean ± SD)	51 ± 43.9	739 ± 598		< 0.0001
Montreal classification* of UC extent, n (%)				
E1	3 (15)	4 (26.6)		
E2	12 (60)	7 (46.8)		
E3	5 (25)	4 (26.6)		
Montreal classification of severity, n (%)				
S0	20 (100)	0 (0)		
S1	0 (0)	0 (0)		
S2	0 (0)	5 (33.3)		
S3	0 (0)	9 (60)		
Comorbidities, *n* (%)				
Degenerative joint disease	5 (14.3)			
Primary sclerosing cholangitis	4 (11.4)			
Autoimmune hepatitis	3 (8.6)			
Reflux	2 (5.7)			
Asthma	2 (5.7)			
Kidney tumor	1 (2.8)			
Osteoporosis	1 (2.8)			
Medication, *n* (%)				
Biologic treatment	7 (20)			
5-ASA drugs	27 (77.1)			
Thiopurines	5 (14.3)			
Steroids	11 (31.4)			

*BMI* body mass index, *CRP* C-reactive protein

*Based on Satsangi J. 2006

### Gut microbiota diversity

The alpha-diversity of microbial samples was determined based on OTU richness and Shannon entropy. Significant difference was revealed in Shannon entropy between HC and UC_E, as well as between UC_R and UC_E (*P* = 0.0017 and *P* = 0.012, respectively) (Fig. S1A). The mean number of OTUs for HC was 912 (± 157), while UC_E was the least diverse group, with a mean number of OTUs of 800 (± 210).

Using Bray‒Curtis dissimilarity index, the beta-diversity of the studied samples was illustrated, however, the groups did not cluster significantly different from each other (Fig. S1B).

### Taxonomic composition of the microbial community

**Fig. 1:**
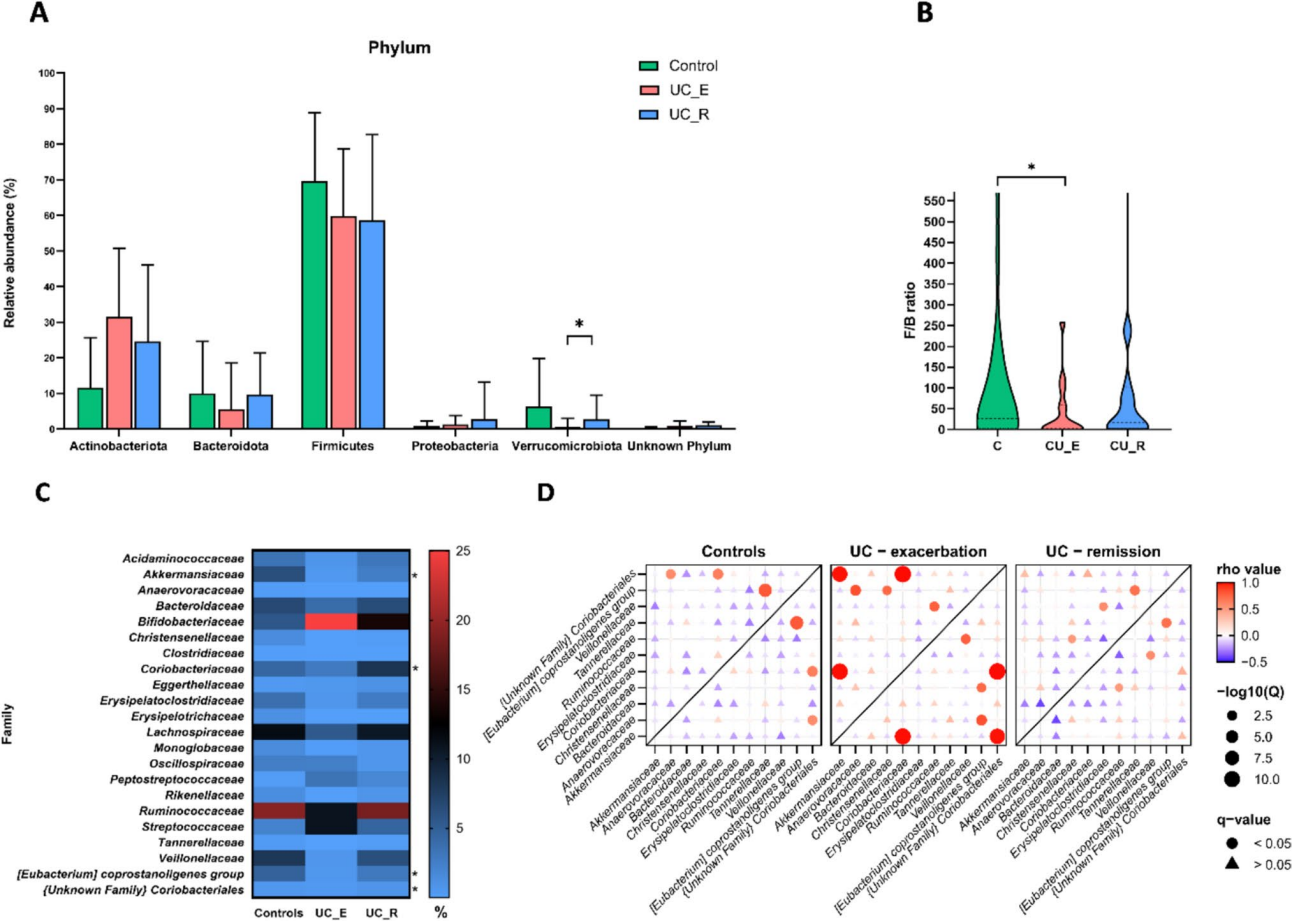
**A** The abundance of the main bacterial phyla in all studied groups. Data shown as the means ± SDs; Mann‒Whitney test with the Benjamini‒Hochberg procedure was used; **B** *Firmicutes*/*Bacteroidetes* ratio. Medians with min and max; Mann‒Whitney test; **C** Abundance of the main bacterial families in the studied groups. Mean abundance (%); Mann‒Whitney test with Benjamini‒Hochberg procedure; **D** Correlation analysis at the bacterial family level in UC patients and healthy controls. Only families primarily differentiated inflammation from remission in UC patients were included. *P < 0.05

Among all identified bacterial taxa, only those presented in at least 50% of the tested fecal samples were selected for further comparative analyses. Thus, at the phylum level, 6 phyla from 14 primarily identified were included (Fig. 1A**, **Table S1). In all groups, *Firmicutes* was the dominant phylum, followed by *Actinobacteria* and *Bacteroidetes*. UC_E patients differed significantly from UC_R and HC only in terms of *Verrucomicrobia* abundance, which was of 0.6%, 2.6% and 6.3%, respectively (*P* = 0.017 and *P* < 0.001, respectively).

Calculated *Firmicutes*/*Bacteroidetes* ratio (*F*/*B*) was much greater among HC (mean ratio 478.1) than among UC patients (mean ratio 129.1), although the difference was not significant (*P* = 0.138), confirming the dysbiosis in UC. A significant difference was observed only between HC and UC_E (*P* = 0.019), but not between UC_E and UC_R (*P* = 0.066; Fig. 1B).

The UC_E was lower enriched than UC_R and HC of *Akkermansiaceae* (0.6% *vs.* 2.6%,* P* = 0.037)*, Coriobacteriaceae* (2.9% *vs.* 8.3%, *P* = 0.028), *Coprostanoligenes* group (0.64% *vs.* 3.04%, *P* = 0.028) and an unknown family from the order *Coriobacteriales* (0.42 *vs.* 0.83, *P* = 0.028)*.* Results of the interaction between bacterial families differentiated UC_E from UC_R were illustrated as a bubble plots, presenting that UC_R is more similar to HC, unlike the UC_E is significantly distinguished by strong positive correlations between particular bacterial families (Fig. 1C, D).

### Gut microbiota associated with remission and exacerbation in UC patients

Being focused particularly on bacterial genus and species in detection taxa significantly associated with the disease, we revealed that 5 genera and 11 bacterial species significantly differentiated UC_E from UC_R (Table S1).

**Fig. 2:**
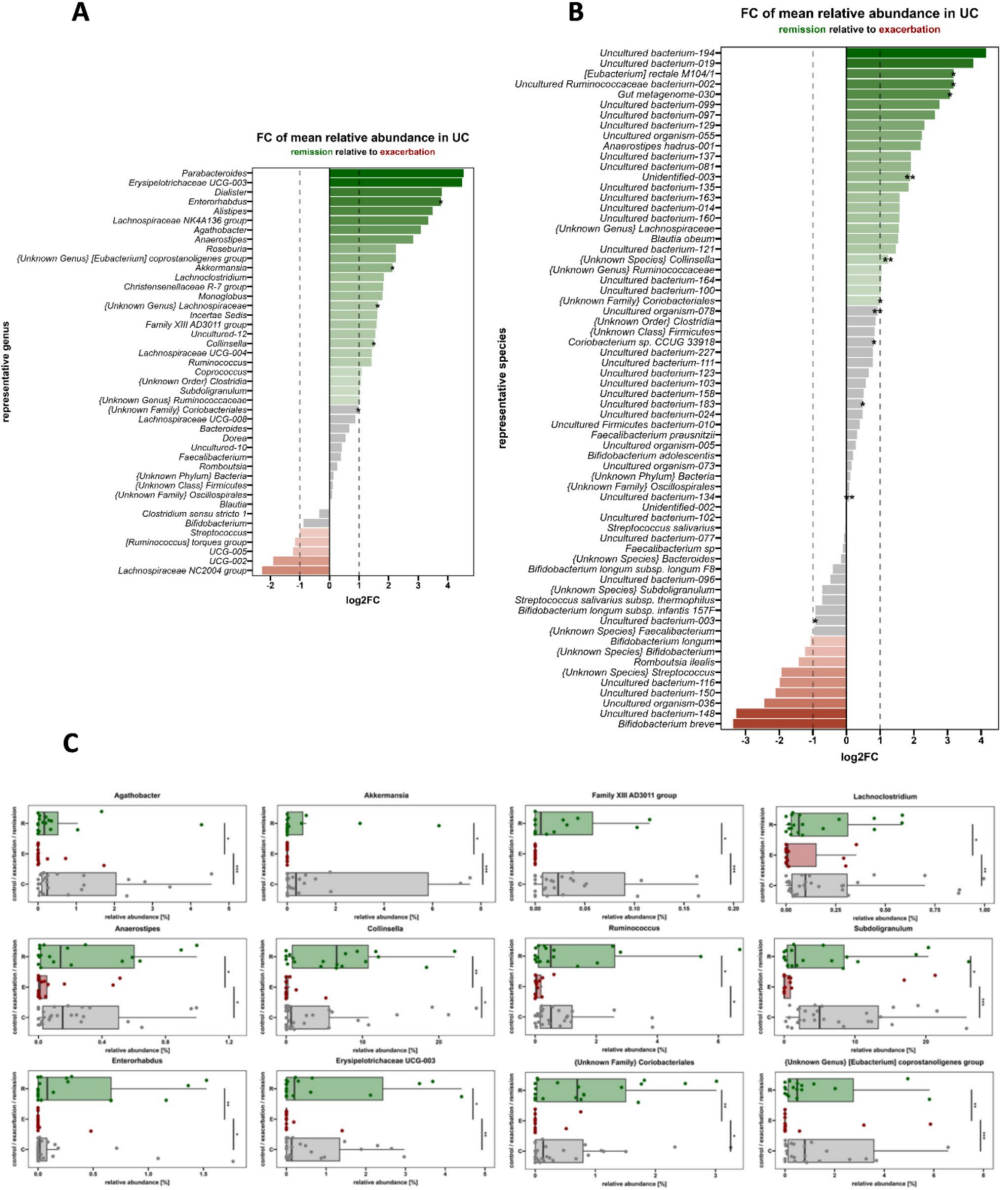
**A** Bacterial genera associated with remission and exacerbation of UC. **B** Bacterial species associated with remission and exacerbation of UC. **C** Abundance of bacterial genera associated with remission. Data shown as medians with min and max. Kruskall‒Wallis test with post hoc Dunn’s test, corrected with Benjamini–Hochberg procedure was used; * *P* < 0.05; ** *P* < 0.01; *** *P* < 0.001

Based on the FC analysis at the genus level, we observed *Parabacteroides* and *Erysipelotrichaceae UCG-003* approximately four times enriched in the UC_R than in UC_E. Furthermore, the richness of *Dialister, Enterorhabdus, Alistipes*, and *Lachnospiraceae NK4A136* was more than three times greater in the UC_R than in HC. However, *Akkermansia*, *Collinsella*, *Enterorhabdus*, *{Unknown Family} Coriobacteriales*, and *{Unknown Genus} Lachnospiraceae* were significantly associated with the UC_R (*P* adj < 0.05; Fig. 2A). The genera *Lachnospiraceae NC2004* group, *Streptococcus*, *Ruminococcus torques* group, *UCG-005*, and *UCG-002* were linked with UC_E.

Nearly 78% of all analyzed bacterial species were unknown or unidentified. Similarly, based on the FC analysis of bacterial species, we observed two various uncultured bacterial species approximately four times more abundant in UC_R than in UC_E (Fig. 2B). Furthermore, the richness of *[Eubacterium] rectale M104/1,* one uncultured *Ruminococcaceae* bacterium*,* and one *gut metagenome* species was more than three times greater in UC_R. A significant association with the UC_R was also detected for *[Eubacterium] rectale M104/1,* an unknown species of *Colinsella,* an unknown family of *Coriobacteriales, Coriobacterium* sp. *CCUG 33918*, and several unknown or uncultured bacteria. *Romboutsia ilealis* and one unknown species of *Streptococcus* were linked with UC_E.

Combining the highest FC (FC ≥ 1.5) and the significant difference in bacterial genera abundance between both, the exacerbation and remission, as well as between exacerbation and healthy controls, we have indicated *Agathobacter*, *Akkermansia*, *Anaerostipes*, *Enterorhabdus, Colinsella, Erysipelotrichaceae UCG-003, Ruminococcus, Subdoligranulum, Lachnoclostridium, Family XIII AD3011 group, Coriobacteriales* and *Coprostanoligenes group* as the potential biomarkers of UC remission (Fig. 2C).

### Fecal metabolites associated with remission and exacerbation in UC patients

All metabolites from 65 fecal samples were identified using GC‒MS analysis. This approach allowed us to detect 349 compounds, of which 115 significantly differentiated UC from HC (*P* < 0.05) (Table S2).

**Fig. 3:**
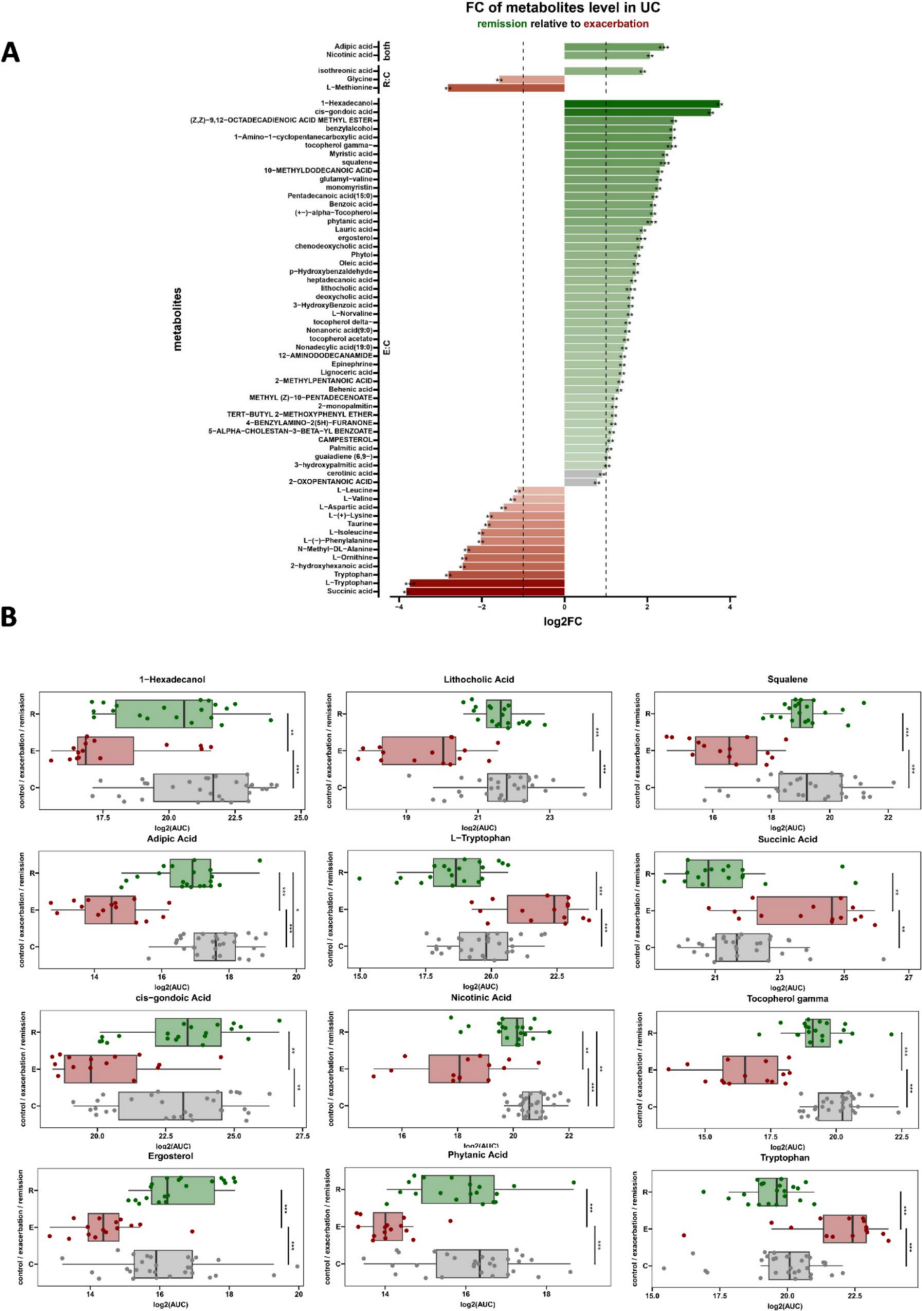
**A** Fecal metabolites associated with remission and exacerbation of UC. Bar plot with different *P* values and FC thresholds; shown are only metabolites *P* adj < 0.01 (**) and |log2FC|> 1 (FC = 2 vertical thresholds). For metabolites grouped according to their differences in levels from the control, “E:C” indicates that metabolite levels are significantly different between the E and C groups, “R:C” indicates that metabolite levels are different between the R and C groups, and “both” indicates that these metabolites are different from the control in both groups (E and R). Data obtained by the Mann‒Whitney test corrected with the Benjamini‒Hochberg procedure; **B** Fecal metabolites most significantly differentiating UC exacerbation from UC remission patients and healthy controls. Boxplots visualizing differences have been included for selected metabolites (which have the highest significance or FC value). Boxplots represent the median values. * *P* < 0.05; ** *P* < 0.01; *** *P* < 0.001

Among the numerous fecal compounds significantly differentiating UC_E from UC_R, only some metabolites also showed a crucial difference in the level between UC_E and HC (61 in total, including 48 compounds associated with UC_R and 23 with UC_E). These metabolites included adipic acid, tocopherol gamma, squalene, phytanic acid, ergosterol and lithocholic acid, whose levels were found to be most significantly (*P* < 0.001) greater in remission. During exacerbation, the L-tryptophan concentration significantly increased (*P* < 0.001). However, based on FC analysis, we revealed that L-tryptophan and succinic acid, which are crucial in UC_E, were almost four times more abundant in this group of patients, while in UC_R, 1-hexadecanol and cis-gondoic acid were almost four times more abundant in this group of individuals (Fig. 3A).

Combining the results of the highest FC (FC > 2) and the significant difference in metabolite content between both, the exacerbation and remission (*P* < 0.01), and between exacerbation and healthy control (*P* < 0.01) groups, we selected compounds that have the potential to be human fecal indicators of UC course. Finally, twelve metabolites met these criteria: 1-hexadecanol, lithocholic acid, squalene, adipic acid, cis-gondoic acid, nicotinic acid, tocopherol gamma, ergosterol and phytanic acid as a indicators of remission, and tryptophan, L-tryptophan, and succinic acid as a biomarkers of exacerbation (Fig. 3B**)**.

### SCFAs

Using ultra-performance liquid chromatography coupled to high-resolution mass spectrometry (UPLC-HESI-HRMS/MS) SCFAs in 65 fecal samples were detected, including acetic acid, propionic acid, butyric acid, n-valeric acid, caproic acid and enanthic acid (Table S3).

The UC_E group was characterized by a significantly lower richness of propionic acid than UC_R and HC, but only before FDR correction (Table S4**, **Fig. S2A). Moreover, UC_E presented significantly lower levels of caproic and enanthic acid in comparison to HC but not to UC_R. On the other hand, the SCFAs concentration in UC_R was generally similar to that in HC, except for that of enanthic acid (*P* < 0.05), the level of which was significantly greater in HC. Principal component analysis (PCA) illustrates that the dissimilarity of the groups is not as evident (Fig. S2B). Correlations between bacterial genera and identified SCFAs are presented on the Fig. S2C.

### Serum metabolites associated with course of UC

Over thousand different metabolites has been found in the serum samples from all individuals included in the study and 418 compounds showed significant differences in amount between the all studied groups (Table S5). Among them, mainly bile acids, amino acids, alcohols, vitamins, fatty acids, esters, carboxylic acids, indoles, xanthines, and their derivatives were identified.

**Fig. 4:**
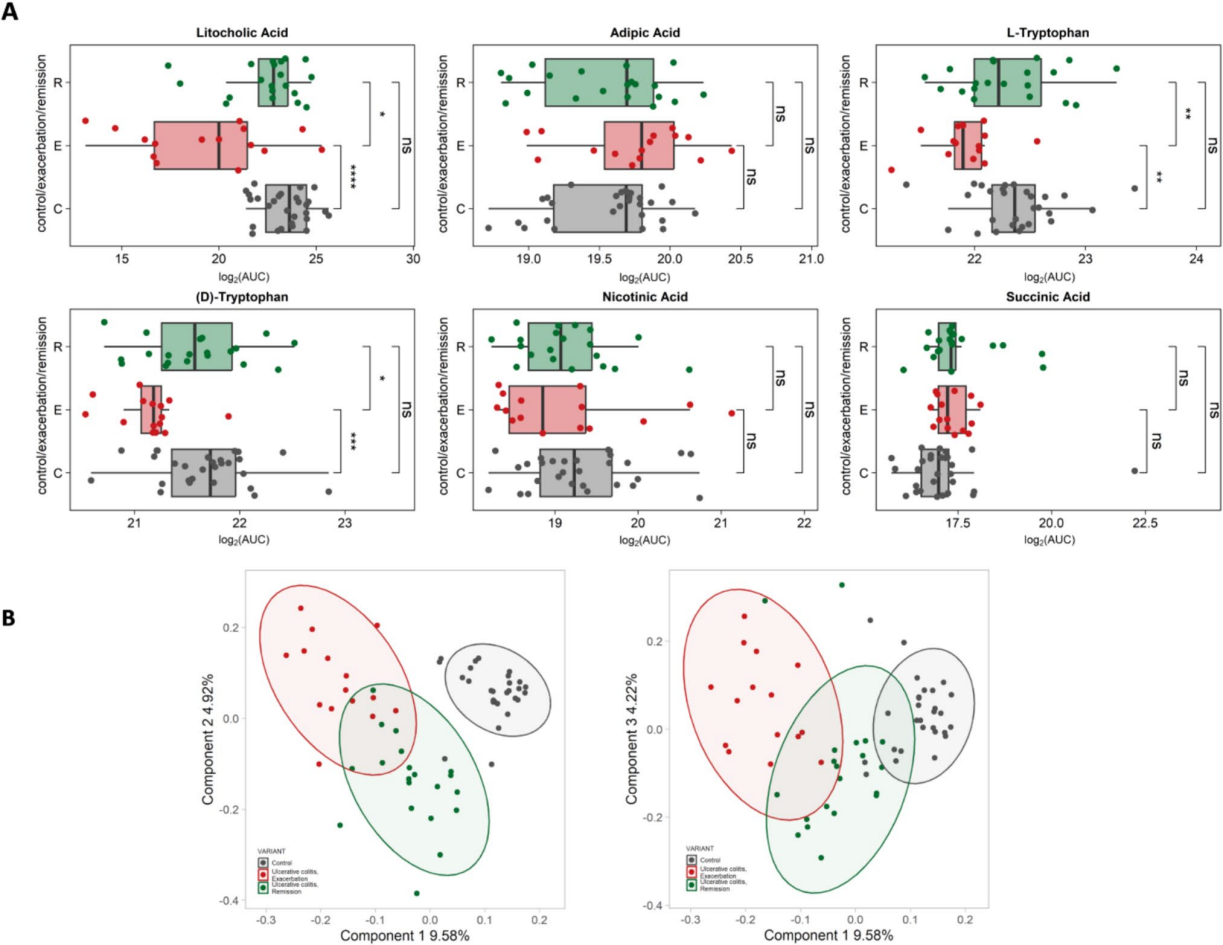
**A** Potential fecal biomarkers of UC course verified in the serum. Boxplots represent the median values. * *P* < 0.05; ** *P* < 0.01; *** *P* < 0.001; **** *P* < 0.0001. **B** PLSDA plots of UC patients and healthy controls based on serum metabolites richness

We have verified the presence of the most significant in UC course fecal metabolites also in the serum. Our results has shown that lithocholic acid, adipic acid, L-tryptophan, tryptophan, nicotinic acid, and succinic acid were found also in the serum. However, among them, only lithocholic acid was observed significantly increased in remission both, in serum and stool (Fig. 4A).

We have observed, based on partial least squares discriminant analysis (PLS-DA), that UC patients in remission are separately grouped from the patients being in the exacerbation state, however still only partly similar, whereas healthy controls are separated from patients, having only common part with patients in remission (Fig. 4B).

**Fig. 5:**
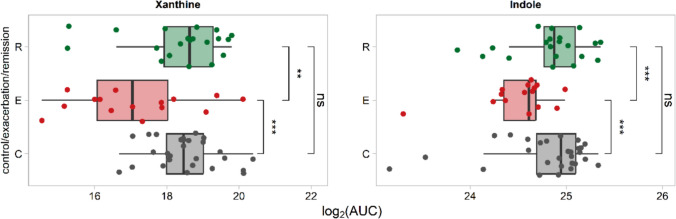
Identified potential serum biomarkers of remission. Boxplots represent the median values. * *P* < 0.05; ** *P* < 0.01; *** *P* < 0.001

Among all identified metabolites in the serum, we have observed xanthine and indole as a potential biomarkers of remission state in UC. Both presented the strongest association with remission state of UC, as well as the significant difference in amount between UC patients in exacerbation and healthy controls **(**Fig. 5**)**.

### Crosstalk between the gut microbiota and metabolites

To understand the relationship between bacterial disturbances and changes in the composition of metabolites, we created a correlation matrice for bacterial genera with metabolites based on calculations using Spearman’s rank correlation test.

**Fig. 6:**
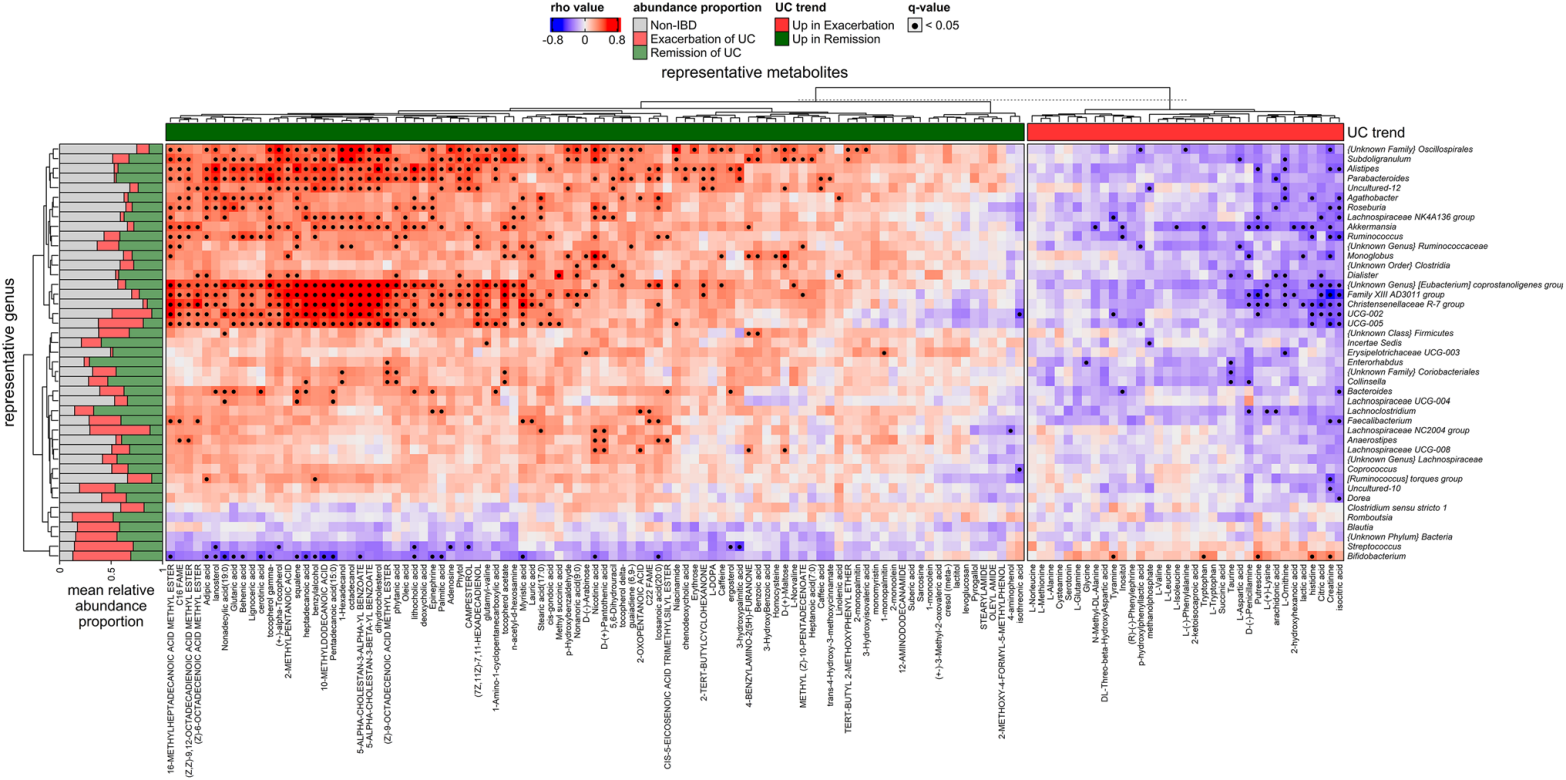
Heatmap summarizing the correlation of the gut microbiota with metabolites in UC remission and exacerbation at the bacterial genus level. The bar plots on the left side show the mean relative abundance proportions of bacterial genera listed on the right side of the plot; on the bottom of the plot, there are significantly different metabolites between remission and exacerbation, *P* < 0.01 (**) and |log2FC|> 1. On the right side of the plot, bacterial genera identified with 50% frequency in the analyzed samples are listed. On the upper side of the plot, significantly increased metabolites are marked in color – in remission (green) and exacerbation (red); statistical significance is marked with a dot in the square (for adjusted *P* value < 0.05). Blue squares indicate negative (inverse) correlation, and red squares indicate positive correlation; shading of the squares indicate the magnitude of the correlation, which was calculated using Spearman’s rank correlation test

On the heatmaps (Fig. 6 and Fig. S3), a very clear, highly positive correlation block of metabolites significantly increased in the remission group is visible. This confirmed our partial findings of metabolic biomarkers of remission presented on Fig. 3B, such as adipic acid, tocopherol gamma, squalene, 1-hexadecanol. We also noted specific bacteria composition among UC_R, such as the *Coprostanoligenes* group {unknown genus}, *Christensenellaceae* R-7 group, *Family XIII AD3011* group and others, as *UCG-002* and *UCG-005.* The same relationship was observed between beneficial *Akkermansia*, which was significantly enriched in UC_R (*P* adj < 0.05) and tocopherol gamma, 1-hexadecanol; as well as between *Colinsella* (also enriched in UC_R, *P* adj < 0.05), 1-hexadecanol and phytanic acid, similar to the findings for *Colinsella* and the {unknown family} *Coriobacteriales*, with phytanic acid and 1-hexadecanol.

On the other hand, for the same set of bacteria (*Coprostanoligenes* group {unknown genus}, *Christensenellaceae* R-7 group, Family XIII AD3011 group), which were significantly less abundant (Table S1) among UC_E, there was a separate block of inverse correlations with metabolites that were enriched in UC_E, such as citric acid, isocitric acid, and creatinine, whose levels were increased in UC_E. L-tryptophan and tryptophan were the most significantly associated with UC_E (Fig. 3B), and these parameters exhibited an inverse correlation with *Akkermansia* abundance.

The strongest positive correlation between bacteria and SCFAs was observed for enanthic acid and *Oscillospirales* {unknown family}, *UCG-002*, *Subdoligranulum*, and *Family XIII AD3011* (Fig. S2C). Moreover, caproic acid, which was significantly more abundant in controls than in UC_E and more abundant in UC_R than in UC_E was positively correlated with *Akkermansia*, *[Eubacterium] coprostanoligenes* {unknown genus}, *UCG-002*, and the *Family XIII AD3011* group, which were associated with remission. The same bacterial genera were correlated with n-valeric acid.

## Discussion

Although dysbiosis has been characterized more thoroughly in UC patients than in healthy individuals, the differences in microbial structure between patients with active disease and patients in remission are unclear [18, 23–25]. Identifying differences in the composition of gut microbiota and microbiota-derived metabolites specific to the remission and flare states in UC could not only improve our understanding of disease pathogenesis but also support the development of new therapeutic options.

In presented study, we followed the hypothesis that gut microbiota and microbiota-associated metabolites could drive the UC disease course.

We confirmed gut dysbiosis in UC patients. The highest alpha-diversity value was found for controls, followed by remission and exacerbation of UC [26] (Fig. S1A). Our results indicated important complex differences in microbial and metabolite composition due to disease activity. Active UC compared with remission, was associated with a lower abundance of *Verrucomicrobiota*. The *F*/*B* ratio in UC_E was lower than that in HC (*P* = 0.019). Moreover, UC_E were characterized by significantly lower richness of the *Akkermansiaceae, Coriobacteriaceae*, and *Coprostanoligenes* groups and an unknown family from the order *Coriobacteriales* than UC_R and HC. Finally, we identified the following genera as bacterial biomarkers of remission in our cohort of Polish UC patients: *Family XIII AD3011 group*, *Lachnoclostridium*, *Subdoligranulum*, *[Eubacterium] coprostanoligenes*, *Agathobacter*, and *Akkermansia*. These bacteria were markedly more abundant in the healthy control group than in the remission, with the lowest abundance occurring in the exacerbation group. After function analysis of all of them based on available databases, we have observed that those bacteria are beneficial microbes, involved in the following processes in the intestine, as strengthening of the intestinal barrier, mucus layer formation, regulation of microbial balance, protection against pathogens, what lead to enhancing the anti-inflammatory impact (MiMeDatabase; Data Repository for Human Gut Microbiota). The strong positive correlation between the bacterial families *Akkermansiaceae* and *Coriobacteriaceae* appears to be particularly important during the flare state of UC, in contrast to the remission phase or healthy individuals (Fig. 1D).

To date, metagenomic data have revealed a correlation between a reduced abundance of *Akkermansia* (particularly *A. muciniphila*) and diseases, such as IBD, obesity, and diabetes [27]. *Akkermansia,* a Gram-negative obligate anaerobe belonging to the *Verrucomicrobiota* phylum, possesses great beneficial activity in the human intestine because it produces important SCFAs, such as acetate and propionate, as a consequence of host-glycan degradation [28]. Recently, this bacterium was identified as an immunomodulatory probiotic that can accelerate inflammation, which is consistent with ongoing clinical research [29, 30]. Our results confirmed a positive correlation between the abundance of the *Akkermansia* genus and the amount of caproic, n-valeric, enanthic, propionic and acetic acids in the stool samples. The same correlation was found for these SCFAs and *Agathobacter*, as well as one unknown genus of the *coprostanoligenes group [Eubacterium]*. In particular, a strong positive correlation was observed between the *Family XIII AD3011 group* and enanthic, n-valeric and caproic acids (Fig. S2C). On the other hand, the levels of two SCFAs — acetic and propionic — were substantially greater in the remission group and healthy controls than in the exacerbation group, although these differences were not statistically significant (Fig. S2A). To our surprise, none of the identified SCFAs showed a strong correlation with UC remission. However, studies demonstrate that butyrate is one of the most important SCFAs, and its role is to serve as a source of energy for colonocytes, strengthen the tightness of the intestinal barrier and to calm inflammation, which has a vital effect on the human organism [31]. Hence, we expected a protective effect of butyrate on UC in our study.

We also confirmed, that gut microbial dysbiosis and specific bacterial shifts in active UC have consequences for the composition of fecal and serum metabolites. Among the fecal metabolites associated with remission we detected adipic acid, cis-gondoic acid, ergosterol, lithocholic acid, phytanic acid, squalene, tocopherol gamma, nicotinic acid, and 1-hexadecanol. The fact that the levels of these metabolites are not dependent on the inflammation marker (CRP) supports the hypothesis that they may serve as potential indicator metabolites in the course of UC, closely related to the composition of the intestinal microbiota (Table S6). Whereas L-tryptophan, succinic acid, and tryptophan levels in feces were strongly correlated with exacerbation. Tryptophan (Trp) is an essential, exogenous amino acid in humans, and its biotransformation plays a central role in microbiota-host crosstalk. Alterations in Trp metabolism in IBD have been already described [32]. The role of microbiota in tryptophan metabolism is significant because microbial enzymes are involved in this process. One of the main transformation pathways of Trp is the kynurenine pathway (KP), during which nicotinic acid is produced. Interestingly, in our study Trp is accumulated in the exacerbation state compared to the remission, while nicotinic acid (the end product of KP) was found to be accumulated in patients in remission phase. Both of these compounds are negatively linked to each other, which indicates that the augmentation of nicotinic acid (vitamin B3) and tocopherol gamma (vitamin E) supports the maintenance of remission.

We decided to more focus on the bacterial branch of Trp metabolism, particularly the pathway involving the enzyme tryptophanase (*TnaA*, EC 4.1.99.1), which is produced by certain beneficial gut bacteria. In this pathway finally indoles are produced. Indole derivatives (e.g., indole-3-acetic acid) are able to inhibit pro-inflammatory cytokines through suppression of NF-κB signaling [33]. As a result, indole metabolites strengthen tight junctions and stimulate mucin production, reducing bacterial translocation [34]. In our study, an increased level of indoles we have found in the serum of UC patients being in remission state **(**Table S5**; **Fig. 5**)**. Moreover, we inferred the presence of the *TnaA* enzyme from bacterial community profiles using a PICRUSt-based functional pathway prediction analysis in the CLC software. The results revealed highest presence of tryptophanase in the control group and patients in remission (6%) compared to patients in exacerbation state (1.5%), which explains the levels difference. This observations confirm an important role of gut bacteria in Trp metabolism and in disease course.

Our attention was also drawn to another metabolite, serum lithocholic acid (LCA), which was found to be significantly associated with UC remission (Fig. 5). Lithocholic acid is a secondary bile acid, produced with the participation of gut beneficial bacteria. Recent studies revealed its possible anti-inflammatory role and impact in mucosal barrier improvement [35, 36]. The anti-inflammatory effect of LCA is based on the activation of TGR5/PKA and inhibition of the NF-κB pathways [37]. In addition, LCA induces the expression of proteins, such as occludin, which strengthen tight junctions between enterocytes. Studies suggest, that increased LCA, produced by beneficial bacteria, may mitigate UC [38, 39]. In our study among positively correlated bacteria with LCA was for example *Akkermansia*, *Family XIII AD3011 group*, and *Christensenellaceae R-7 group.* These conclusions confirm our belief that LCA has a potential to be a candidate marker of UC remission, as we observed in our Polish patients.

Our study also provided a huge amount of data on the correlation of intestinal bacteria with fecal metabolites (Fig. 6). This is certainly material for further research, but for now it has allowed us to clearly distinguish exacerbation from remission in UC, to better understand the pathogenesis of the disease itself, but also its course. A strong positive correlation was found between tocopherol gamma and bacterial predictors of remission, such as *Akkermansia*, the *[Eubacterium] coprostanoligenes group*, and the *Family XIII AD3011 group* (*r* > 0.5, *P* < 0.05). Furthermore, the elevated level of ergosterol, which is a provitamin D2, was found among UC patients in remission. The nearly two-fold greater level of ergosterol in the remission group than in the exacerbation group may be a result of the decreased amount of sterol 24-C-methyltransferase in this group of patients, an enzyme involved in the steroid biosynthesis pathway (PICRUSt, enzyme number 2.1.1.41; MetaCyc).

The strength of our research is the precise selection of criteria for including participants into the study, supported by the results of statistical comparisons of the demographic and clinical data between groups.

We are also aware of the limitations of our study. First, metagenomic analysis at the genus level did not allow for the detection of numerous well-known species, such as *Akkermansia muciniphila*. On the other hand, the share of unknown and uncultured bacterial species was dominant, which shows that a major part of the entire intestinal microbiota remains unknown. Some of them (e.g., *unidentified-003* and *uncultured bacterium-183*) showed significant differences between remission and exacerbation states in our study, which encouraged us to investigate these previously uncultured bacteria.

We identified specific microbial and metabolic signatures associated with remission and flare in UC, which may contribute to progress in understanding the pathophysiology of the disease. These signatures have also been partially observed in a previous study [40].

The results of our research revealed a number of complex relationships between the broadly defined bacterial microbiota and metabolome across different clinical courses of UC. Although they do not fully explain the mechanisms that determine disease progression, they clearly define the bacterial and metabolic characteristics of remission in UC.

## Conclusion

This is the first comprehensive study presenting significant differences between UC courses—remission state and flare in the field of gut microbiota composition and metabolites. The results definitely help in understanding the possible mechanism of remission in patients with UC and may constitute a milestone in developing UC therapy by maintaining constant remission of the disease in future.

## Supplementary Information

Below is the link to the electronic supplementary material.

Supplementary file1 (Figure S1) (TIF 3151 KB)

Supplementary file2 (Figure S2) (TIF 3635 KB)

Supplementary file3 (Figure S3) (JPG 11973 KB)

Supplementary file4 (Legend of Supplements) (DOCX 16 KB)

Supplementary file5 (Supplementary Tables S1-S6) (XLSX 817 KB)

## Abbreviations

BMI: Body mass index
*F/B*: *Firmicutes/Bacteroidetes* Ratio
FC: Fold-change
GC/MS: Gas chromatography combined with mass spectrometry
IBD: Inflammatory bowel diseases
LC: Liquid chromatography
LCA: Lithocholic acid
OTU: Operational taxonomic unit
PCA: Principal component analysis
SCFA: Short-chain fatty acid
UC: Ulcerative colitis
UPLC-HESI-HRMS/MS: Ultra-performance liquid chromatography coupled to high-resolution mass spectrometry

## References

[1] Graham DB, Xavier RJ. Pathway paradigms revealed from the genetics of inflammatory bowel disease. Nature. 2020;578(7796):527–39. 32103191 10.1038/s41586-020-2025-2 PMC7871366

[2] Spencer EA, Helmus D, Telesco S, et al. Inflammatory bowel disease clusters within affected Sibships in Ashkenazi Jewish multiplex families. Gastroenterology. 2020;159(1):381–2. 32199878 10.1053/j.gastro.2020.03.023

[3] Kobayashi T, Siegmund B, Le Berre C, et al. Ulcerative colitis. Nat Rev Dis Prim. 2020;6(1):1–20. 32913180 10.1038/s41572-020-0205-x

[4] Eisenstein M. Ulcerative colitis: towards remission. Nature. 2018;563(7730):S33. 30405234 10.1038/d41586-018-07276-2

[5] Lane ER, Zisman TL, Suskind DL. The microbiota in inflammatory bowel disease: current and therapeutic insights. J Inflamm Res. 2017;10:63–73. 28652796 10.2147/JIR.S116088 PMC5473501

[6] Magarian Blander J, Longman RS, Iliev ID, et al. Regulation of inflammation by microbiota interactions with the host HHS Public access author manuscript. Nat Immunol. 2017;18(8):851–60. 28722709 10.1038/ni.3780 PMC5800875

[7] Mańkowska-Wierzbicka D, Stelmach-Mardas M, Gabryel M, et al. The effectiveness of multi-session FMT treatment in active ulcerative colitis patients: a pilot study. Biomedicines. 2020;8(8):268. 10.3390/biomedicines8080268. 32756350 10.3390/biomedicines8080268 PMC7459721

[8] Mańkowska-Wierzbicka D, Zuraszek J, Wierzbicka A, et al. Alterations in gut microbiota composition in patients with COVID-19: A pilot study of whole hypervariable 16S rRNA gene sequencing. Biomedicines. 2023;11(2):367. 10.3390/biomedicines11020367. 36830905 10.3390/biomedicines11020367 PMC9953267

[9] de Jong RJ, Ohnmacht C. Defining dysbiosis in inflammatory bowel disease. Immunity. 2019;50(1):8–10. 30650382 10.1016/j.immuni.2018.12.028

[10] Britton GJ, Contijoch EJ, Mogno I, et al. Microbiotas from humans with inflammatory bowel disease alter the balance of gut Th17 and RORγt+ regulatory t cells and exacerbate colitis in mice. Immunity. 2019;50(1):212-224.e4. 30650377 10.1016/j.immuni.2018.12.015 PMC6512335

[11] Cho S, Stroup BM, Britto SL, et al. Increased number of children in households may protect against inflammatory bowel disease. Pediatr Res. 2023;93(3):535–40. 10.1038/s41390-022-02149-x. 35701607 10.1038/s41390-022-02149-x

[12] Zakerska-Banaszak O, Zuraszek-Szymanska J, Eder P, et al. The role of host genetics and intestinal microbiota and metabolome as a new insight into IBD pathogenesis. Int J Mol Sci. 2024;25(17):9589. 10.3390/ijms25179589. 39273536 10.3390/ijms25179589 PMC11394875

[13] Walker AW, Sanderson JD, Churcher C, et al. High-throughput clone library analysis of the mucosa-associated microbiota reveals dysbiosis and differences between inflamed and non-inflamed regions of the intestine in inflammatory bowel disease. BMC Microbiol. 2011;11:7. 21219646 10.1186/1471-2180-11-7 PMC3032643

[14] Zakerska-Banaszak O, Tomczak H, Gabryel M, et al. Dysbiosis of gut microbiota in Polish patients with ulcerative colitis: a pilot study. Sci Rep. 2021;11(1):2166. 10.1038/s41598-021-81628-3. 33495479 10.1038/s41598-021-81628-3 PMC7835370

[15] Gallagher K, Catesson A, Griffin JL, et al. Metabolomic analysis in inflammatory bowel disease: a systematic review. J Crohns Colitis. 2021;15(5):813–26. 10.1093/ecco-jcc/jjaa227. 33175138 10.1093/ecco-jcc/jjaa227

[16] Krautkramer KA, Kreznar JH, Romano KA, et al. Diet-microbiota interactions mediate global epigenetic programming in multiple host tissues. Mol Cell. 2016;64(5):982–92. 10.1016/j.molcel.2016.10.025. 27889451 10.1016/j.molcel.2016.10.025 PMC5227652

[17] Krautkramer KA, Fan J, Bäckhed F. Gut microbial metabolites as multi-kingdom intermediates. Nat Rev Microbiol. 2021;19(2):77–94. 32968241 10.1038/s41579-020-0438-4

[18] Lloyd-Price J, Arze C, Ananthakrishnan AN, et al. Multi-omics of the gut microbial ecosystem in inflammatory bowel diseases. Nature. 2019;15:22. 10.1038/s41586-019-1237-9 PMC6650278 31142855

[19] Halfvarson J, Brislawn CJ, Lamendella R, et al. Dynamics of the human gut microbiome in inflammatory bowel disease. Nat Microbiol. 2017;2:17004. 28191884 10.1038/nmicrobiol.2017.4 PMC5319707

[20] Quast C, Pruesse E, Yilmaz P, et al. The SILVA ribosomal RNA gene database project: improved data processing and web-based tools. Nucleic Acids Res. 2013;41(D1):D590-6. 23193283 10.1093/nar/gks1219 PMC3531112

[21] Tsugawa H, Cajka T, Kind T, et al. MS-DIAL: data-i ndependent MS/MS deconvolution for comprehensive metabolome analysis. Nat Methods. 2015;12(6):523–6. 10.1038/nmeth.3393. 25938372 10.1038/nmeth.3393 PMC4449330

[22] Hughes G, Cruickshank-Quinn C, Reisdorph R, et al. MSPrep-summarization, normalization and diagnostics for processing of mass spectrometry-based metabolomic data. Bioinformatics. 2014;30(1):133–4. 10.1093/bioinformatics/btt589. 24174567 10.1093/bioinformatics/btt589 PMC3866554

[23] Caruso R, Lo BC, Núñez G. Host–microbiota interactions in inflammatory bowel disease. Nat Rev Immunol. 2020;20(7):411–26. 32005980 10.1038/s41577-019-0268-7

[24] Vestergaard MV, Allin KH, Eriksen C, et al. Gut microbiota signatures in inflammatory bowel disease. United Eur Gastroenterol J. 2024;12(1):22–33. 10.1002/ueg2.12485 PMC10859715 38041519

[25] Pittayanon R, Lau JT, Leontiadis GI, et al. Differences in Gut microbiota in patients with vs without inflammatory bowel diseases: a systematic review. Gastroenterology. 2020;158(4):930-946.e1. 31812509 10.1053/j.gastro.2019.11.294

[26] Maldonado-Arriaga B, Sandoval-Jiménez S, Rodríguez-Silverio J, et al. Gut dysbiosis and clinical phases of pancolitis in patients with ulcerative colitis. Microbiologyopen. 2021;10(2):e1181. 33970546 10.1002/mbo3.1181 PMC8087925

[27] Rodrigues VF, Elias-Oliveira J, Pereira ÍS, et al. Akkermansia muciniphila and Gut immune system: a good friendship that attenuates inflammatory bowel disease, obesity, and diabetes. Front Immunol. 2022;13:934695. 35874661 10.3389/fimmu.2022.934695 PMC9300896

[28] Kim S, Shin YC, Kim TY, et al. Mucin degrader Akkermansia muciniphila accelerates intestinal stem cell-mediated epithelial development. Gut Microbes. 2021;13(1):1–20. 33678130 10.1080/19490976.2021.1892441 PMC7946046

[29] Bian X, Wu W, Yang L, et al. Administration of Akkermansia muciniphila ameliorates dextran sulfate sodium-induced ulcerative colitis in mice. Front Microbiol. 2019;10:2259. 31632373 10.3389/fmicb.2019.02259 PMC6779789

[30] Zheng M, Han R, Yuan Y, et al. The role of Akkermansia muciniphila in inflammatory bowel disease: current knowledge and perspectives. Front Immunol. 2023;13:1089600. 36685588 10.3389/fimmu.2022.1089600 PMC9853388

[31] Hodgkinson K, El Abbar F, Dobranowski P, et al. Butyrate’s role in human health and the current progress towards its clinical application to treat gastrointestinal disease. Clin Nutr. 2023;42(2):61–75. 10.1016/j.clnu.2022.10.024. 36502573 10.1016/j.clnu.2022.10.024

[32] Agus A, Planchais J, Sokol H. Gut microbiota regulation of tryptophan metabolism in health and disease. Cell Host Microbe. 2018;23(6):716–24. 29902437 10.1016/j.chom.2018.05.003

[33] Roager HM, Licht TR. Microbial tryptophan catabolites in health and disease. Nat Commun. 2018;9:3294. 10.1038/s41467-018-05470-4. 30120222 10.1038/s41467-018-05470-4 PMC6098093

[34] Liu G, Lu J, Sun W, et al. Tryptophan supplementation enhances intestinal health by improving gut barrier function, alleviating inflammation, and modulating intestinal microbiome in lipopolysaccharide-challenged piglets. Front Microbiol. 2022;13: 919431. 10.3389/fmicb.2022.919431. 35859741 10.3389/fmicb.2022.919431 PMC9289565

[35] Hou M, Song P, Chen Y, et al. Bile acids supplementation improves colonic mucosal barrier via alteration of bile acids metabolism and gut microbiota composition in goats with subacute ruminal acidosis (SARA). Ecotoxicol Environ Saf. 2024;12(287): 117313. 10.1016/j.ecoenv.2024.117313. 10.1016/j.ecoenv.2024.117313 39536567

[36] Herrera-deGuise C, Varela E, Sarrabayrouse G, et al. Gut microbiota composition in long-remission ulcerative colitis is close to a healthy gut microbiota. Inflamm Bowel Dis. 2023;29(9):1362–9. 37655859 10.1093/ibd/izad058

[37] Li H, Guo X, Liu X, et al. Protective effect of a cocktail of lactic acid bacteria on DSS-induced ulcerative colitis via activating the TGR5/PKA pathway. Food Biosci. 2024;62: 105326. 10.1016/j.fbio.2024.105326.

[38] Thomas JP, Modos D, Rushbrook SM, et al. The emerging role of bile acids in the pathogenesis of inflammatory bowel disease. Front Immunol. 2022;13: 829525. 10.3389/fimmu.2022.829525. 35185922 10.3389/fimmu.2022.829525 PMC8850271

[39] Gao P, Rinott E, Dong D, et al. Gut microbial metabolism of bile acids modifies the effect of Mediterranean diet interventions on cardiometabolic risk in a randomized controlled trial. Gut Microbes. 2024;16(1):2426610. 10.1080/19490976.2024.2426610. 39535126 10.1080/19490976.2024.2426610 PMC11567240

[40] Barberio B, Facchin S, Patuzzi I, et al. A specific microbiota signature is associated to various degrees of ulcerative colitis as assessed by a machine learning approach. Gut Microbes. 2022;14(1):2028366. 35129058 10.1080/19490976.2022.2028366 PMC8820804

