# Supplementary figures and images for: New potential biomarkers of ulcerative colitis and disease course — integrated metagenomic and metabolomic analysis among Polish patients
Source: J Gastroenterol. 2025 Jul 4;60(11):1384–99. doi: 10.1007/s00535-025-02280-6 (PMC12549426; doi:10.1007/s00535-025-02280-6)

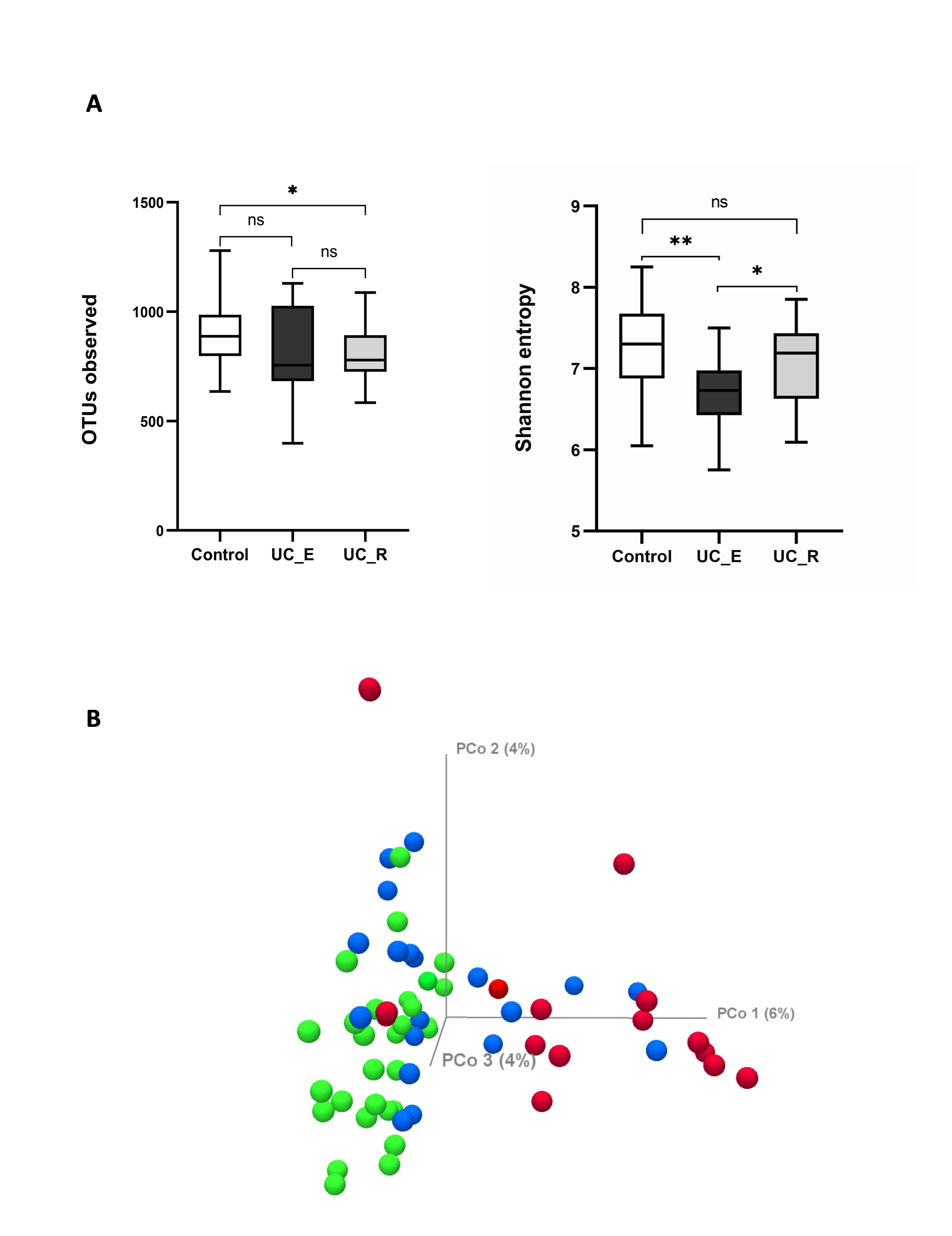

Supplement: Supplementary file 1 — Supplementary file1 (Figure S1) (TIF 3151 KB) [file 535_2025_2280_MOESM1_ESM.tif]

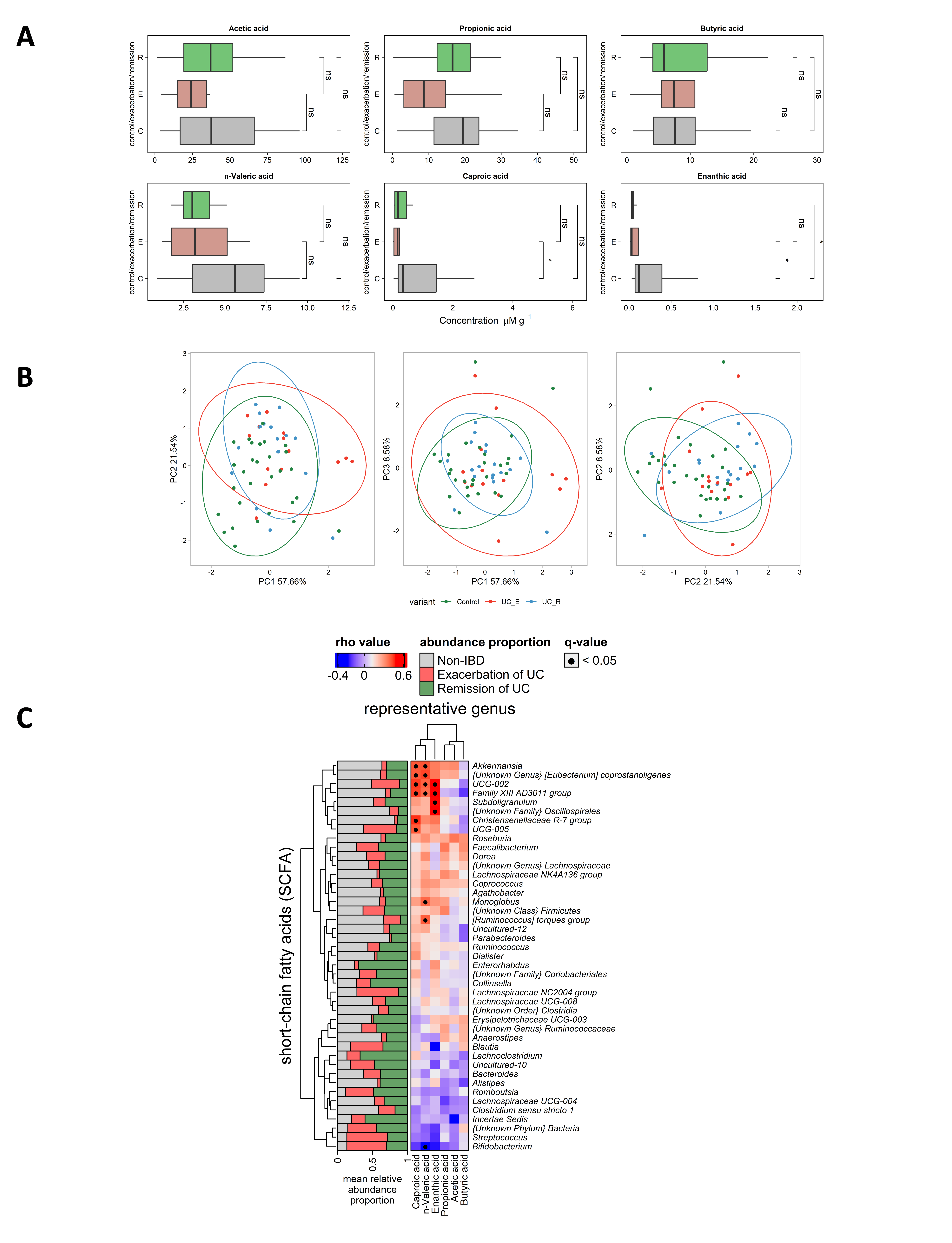

Supplement: Supplementary file 2 — Supplementary file2 (Figure S2) (TIF 3635 KB) [file 535_2025_2280_MOESM2_ESM.tif]
