# Supplementary material for: New potential biomarkers of ulcerative colitis and disease course — integrated metagenomic and metabolomic analysis among Polish patients
Source: J Gastroenterol. 2025 Jul 4;60(11):1384–99. doi: 10.1007/s00535-025-02280-6 (PMC12549426; doi:10.1007/s00535-025-02280-6)
Supplement: Supplementary file 4 — Supplementary file4 (Legend of Supplements) (DOCX 16 KB) [file 535_2025_2280_MOESM4_ESM.docx]

**Legends of Supplementary Data:**

**Supplementary Tables:**

**Table S1.** Bacterial abundance at each taxonomic level filtered by 50% frequency in studied groups.

**Table S2.** Metabolites differentiating UC patients from healthy controls.

**Table S3.** Details of PRM method for quantification of SCFA.

**Table S4.** The summary statistics of SCFA analysis.

**Table S5.** The comparison of serum metabolites in groups.

**Table S6.** Spearman correlation analysis between crucial metabolites and inflammation.

**Supplementary Figures:**

**Figure S1. (A) Alpha diversity as OTU richness (left) and Shannon entropy (right) in all analysed groups.** Data represent the medians with min and max; ns – not significant; * P < 0.05; ** P < 0.01; unpaired Student’s t test; **(B)** **The beta diversity of the analysed groups of samples was determined via principal coordinate analysis (PCoA) with a Bray‒Curtis dissimilarity index**. The colours are as follows: red, UC exacerbation; green, control; blue, UC remission.

**Figure S2. (A) The concentrations of SCFAs determined in the faecal samples of UC patients and healthy controls**. Boxplots represent the median values; ns – not significant; * P < 0.05. Wilcoxon rank sum test was used. **(B) PCA plots of UC patients and healthy controls based on SCFAs richness. (C) Heatmap summarizing the correlation of gut bacterial genera with SCFAs.** The bar plots on the left side show the mean relative abundance proportions of bacterial genera listed on the right side of the plot; on the bottom of the plot, all SCFAs identified in the analysed samples are shown. On the right side of the plot, bacterial genera identified with 50% frequency in the analysed samples are listed; statistical significance is marked with a dot in the square (for adjusted P – value < 0.05). Blue squares indicate negative (inverse) correlations, and red squares indicate positive correlations; shading of the squares indicates the magnitude of the correlation, which was calculated using Spearman’s rank correlation test.

**Figure S3. Heatmap summarizing the correlation of the gut microbiota with metabolites in UC remission and exacerbation at the bacterial species level.** The bar plots on the left side show the mean relative abundance proportions of bacterial species listed on the right side of the plot; on the bottom of the plot, there are significantly different metabolites between remission and exacerbation, *P* < 0.01 (**) and |log2FC|>1. On the right side of the plot, bacterial species identified with 50% frequency in the analysed samples are listed. On the upper side of the plot, significantly increased metabolites are marked in colour – in remission (green) and exacerbation (red); statistical significance is marked with a dot in the square (for adjusted *P* – value < 0.05). Blue squares indicate negative (inverse) correlation, and red squares indicate positive correlation; shading of the squares indicate the magnitude of the correlation, which was calculated using Spearman’s rank correlation test.
